# ACC Deaminase Producing Bacteria With Multifarious Plant Growth Promoting Traits Alleviates Salinity Stress in French Bean (*Phaseolus vulgaris*) Plants

**DOI:** 10.3389/fmicb.2019.01506

**Published:** 2019-07-09

**Authors:** Shikha Gupta, Sangeeta Pandey

**Affiliations:** Amity Institute of Organic Agriculture, Amity University, Noida, India

**Keywords:** ACC deaminase, PGPR, salinity stress, siderophore, indole acetic acid

## Abstract

Plant growth promoting rhizobacteria (PGPR) with 1-aminocyclopropane-1-carboxylic acid (ACC) deaminase activity has the potential to promote plant growth and development under adverse environmental conditions. In the present study, rhizobacterial strains were isolated from Garlic (*Allium sativum*) rhizosphere and were screened *in vitro* ACC deaminase activity in DF salt minimal media supplemented with 3 mM ACC. Out of six isolates, two could degrade ACC into α-ketobutyrate, exhibiting ACC deaminase activity producing more than ∼1500 nmol of α-ketobutyrate mg protein^-1^ h^-1^, and assessed for other plant growth promoting (PGP) functions including indole acetic acid production (greater than ∼30 μg/ml), siderophore, Ammonia, Hydrogen cyanide production and inorganic Ca_3_(PO_4_)_2_ (∼85 mg/L) and ZnSO_4_ solubilization. Besides facilitating multifarious PGP activities, these two isolates augmented *in vitro* stress tolerance in response to 6% w/v NaCl salt stress and drought stress (-0.73 Mpa). The strains ACC02 and ACC06 were identified *Aneurinibacillus aneurinilyticus* and *Paenibacillus* sp., respectively on the basis of 16S rDNA gene sequence analysis and were evaluated for growth promoting potential in French bean seedlings under non-saline and salinity stress conditions through pot experiments. The seed bacterization by ACC02 and ACC06 revealed that treatment of plants with bacterial isolates in the form of consortia significantly declined (∼60%) stress stimulated ethylene levels and its associated growth inhibition by virtue of their ACC deaminase activity. The consortia treatment alleviated the negative effects of salinity stress and increased root length (110%), root fresh weight (∼45%), shoot length (60%), shoot fresh weight (255%), root biomass (220%), shoot biomass (425%), and total chlorophyll content (∼57%) of French bean seedlings subjected to salinity stress.

## Introduction

In most of the developing countries, the crop production is majorly hampered by the negative impact of abiotic (environmental stressors) and biotic stress. Plants being immobile means that they are confronted to various kind of stresses such as drought, flooding, salinity, heat, cold, exposure to heavy metals and nutrient deficiency, as well as phytopathogen and pests attack. The increment in synthesis of ethylene from its immediate precursor, ACC, secreted by plants as root exudates, has been found in almost all plants growing under stress conditions (Wang et al., 2013; Liu et al., 2015; Abiri et al., 2017). Ethylene is a plant hormone involved in regulation of various physiological processes of plants, but the climate change induced ethylene production in plants inflicts a significant reduction in plant growth and development and if not monitored properly could result in plant death (Iqbal et al., 2017; Dubois et al., 2018). Therefore, enhancement in ethylene production is an indicator of susceptibility toward various stresses (Glick, 2014; Müller and Munné-Bosch, 2015).

Among various environmental stressors, excessive presence of salts in soil (soil salinity) is one of the major problematic factors responsible for a reduction of plant growth and crop productivity across the globe. It is reported that more than 1000 million hectares of land is affected by salinity throughout the world as per FAO salt affected soil portal. High concentration of salt (NaCl) not only enhanced the stress ethylene production but also induced ion toxicity, oxidative stress, and disturbed the osmotic potential in plants. All physiological processes like respiration, photosynthesis, nitrogen fixation, etc. are affected by the soil salinity resulting in a decrease in farm productivity and yield (Shabala and Munns, 2012; Paul and Lade, 2014; Acosta-Motos et al., 2017). The problem of soil salinity is frequent in arid and semiarid regions, aggravating due to irrational use of chemical fertilizers and improper use of irrigation water (Bharti et al., 2013).

Plant growth promoting rhizobacteria (PGPR) consisting of a group of beneficial bacteria found in the rhizosphere and rhizoplane of the plants, were proven to be the most environmentally friendly and a better alternative to synthetic agrochemicals and other conventional agricultural practices in augmenting growth and stress tolerance in plants, as well as in attaining sustainable agriculture (Shrivastava and Kumar, 2015; Turan et al., 2017; Gouda et al., 2018; Grobelak et al., 2018; Nagargade et al., 2018). They influence the plant growth both directly and indirectly via various mechanisms like nitrogen fixation, production of plant growth hormone (auxins, cytokinin, and gibberellins), solubilization of phosphates and sequestration of iron by production of siderophores (Bhattacharyya and Jha, 2012; Egamberdieva and Lugtenberg, 2014; Shameer and Prasad, 2018).

The major mechanisms utilized by PGP bacteria to reduce the stress includes lowering the level of ethylene via hydrolysing 1-aminocyclopropane-1-carboxylic acid (ACC) by the enzyme ACC deaminase. ACC is the immediate precursor of the hormone ethylene in plants. It is widely reported that certain PGPR possess ACC deaminase enzyme that can degrade ACC to ammonia and α-ketobutyrate, thus reducing the level of ethylene inside the plants (Gamalero and Glick, 2015; Singh et al., 2015; Raghuwanshi and Prasad, 2018). Therefore, the PGPR containing ACC deaminase has the potential to curb the abiotic stress induced ethylene production and its associated adverse effect on plants. There are several published literatures suggesting that plants inoculated with PGPR containing ACC deaminase make the plant more resistant to various stresses like salinity, drought, flood, and against various pathogens (Pourbabaee et al., 2016; Ravanbakhsh et al., 2017; Ghosh et al., 2018; Saikia et al., 2018).

Therefore, the present study was designed to isolate ACC deaminase producing microbes from rhizospheric soil of garlic (*A. sativum*). Other PGP traits like phosphate solubilization, indole acetic acid (IAA) production, HCN production, and zinc solubilization were also estimated for these isolates. Further, evaluation of plant growth promotion was done in French bean (*Phaseolus vulgaris* L.) variety (*Akra Komal*) plants by bio-priming seeds with individual isolates and consortia of isolates under normal conditions as well as in salinity stress conditions.

## Materials and Methods

### Collection of Rhizospheric Soil Sample

The rhizospheric soil samples of *A. sativum* (garlic), member of *Liliaceae* family was collected during the months of February–March 2018 from the four sites of agricultural farm of *A. sativum* situated at Amity University Uttar Pradesh, India (28.3239°N 77.1959°E), employing organic cultivation practices. Five crop plants were uprooted along with the soil from each site and brought to the laboratory in the zip lock bags for further analysis. The non-rhizospheric soil, as well as large soil aggregates, were removed and soil adhered to the roots were separated from each plant, and mixed together to form one composite pool of rhizospheric soil sample.

### Isolation of Rhizobacteria and Qualitative Estimation of ACC Deaminase Activity

The rhizobacteria were isolated from the soil sample by serial dilution technique in Luria-Bertani (LB) medium (g/L: peptone 10 g; yeast extract 5 g; NaCl 10 g) supplemented with 1.5% agar. 0.1 mL of appropriate dilutions of the sample was plated on LB agar plates and incubated for 24 h at 28°C. The morphologically distinct colonies were screened for ACC deaminase activity on the sterile minimal DF (Dworkin and Foster) salts media (DF salts per liter: 4.0 g KH_2_PO_4_, 6.0 g Na_2_HPO_4_, 0.2 g MgSO_4_.7H_2_O, 2.0 g glucose, 2.0 g gluconic acid and 2.0 g citric acid with trace elements: 1 mg FeSO_4_.7H_2_O, 10 mg H_3_BO_3_, 11.19 mg MnSO_4_.H_2_O, 124.6 mg ZnSO_4_.7H_2_O, 78.22 mg CuSO_4_.5H_2_O, 10 mg MoO_3_, pH 7.2) amended with 3 mM ACC instead of (NH_4_)_2_SO_4_ as sole nitrogen source (Dworkin and Foster, 1958; Penrose and Glick, 2003). The inoculated plates were incubated at 28°C for 3 days and growth was monitored on a daily basis. Colonies growing on the plates were taken as ACC deaminase producers and were purified by sub culturing the isolates.

### Quantification of ACC Deaminase Activity and Confirmation by Fourier Transform Infrared Spectra Analysis

The quantitative assessment of ACC deaminase activity was done spectrophotometrically in terms of α-ketobutyrate production at 540 nm by comparing with the standard curve of α-ketobutyrate, which ranged from 0.1 to 1.0 μmol (Honma and Shimomura, 1978). The protein estimation was done as per Bradford methodology (Bradford, 1976). One unit of ACC deaminase activity was expressed as the amount of α-ketobutyrate liberated in nmol per milligram of cellular protein per hour.

Further, the verification of α-ketobutyrate liberation from a deamination reaction of sole nitrogen source ACC, in DF salt minimal media by isolates with maximum ACC deaminase activity was done as potassium bromide (KBr) cell pellet with the help of Fourier-transform infrared spectroscopy (FTIR) in a FTIR-6700 spectrometer (Nicolet, United States).

### Indole Acetic Acid Production by Rhizobacterial Isolates

The rhizobacterial strains were inoculated in LB medium amended with 5 mM tryptophan and incubated in orbital shaker for 7 days at 28°C at 200 rpm. The IAA production was assessed by the colorimetric method using Salkowski reagent (0.5M FeCl3 + 70% perchloric acid). Development of red color (which indicates the formation of indolic compounds) with addition of Salkowski reagent and cell free culture supernatant (4:1) was measured by UV–vis spectrophotometer at 530 nm (Gordon and Weber, 1951). The concentration of IAA can be determined with a standard curve of pure indole-3-acetic acid (IAA, Hi-media) ranging between 0 and 100 μg mL^-1^.

### Optimization of Indole Acetic Acid Production by Selected ACC Deaminase Producing Isolates

The optimization of IAA production was done based on two parameters, i.e., incubation time and Tryptophan concentration. The overnight grown bacterial culture was inoculated in LB medium supplemented with 5 mM tryptophan. The aliquots of 5 ml bacterial culture were each withdrawn at 24-h intervals for up to 168-h and evaluated for IAA production. Further, to assess the effect of substrate concentration on IAA production, the LB medium was amended with a different concentration of L-tryptophan, which ranged from 0 to 15 mg/ml, and analyzed for IAA production using Salkowski method.

### Phosphate Solubilization by Rhizobacterial Isolates

The ACC utilizing isolates were spot inoculated onto Pikovaskya’s agar medium (Hi-media) amended with 2% (w/v) tricalcium phosphate (TCP) and incubated at 28°C for 4 days. The development of clear zones around the colonies were considered as positive phosphate solublizers. The phosphate solubilizing index (PSI) was calculated by using following formula,

$$\mathrm{PSI}\;=\;\frac{\mathrm{Colony}\;\mathrm{diameter}\;+\;\mathrm{Halozone}\;\mathrm{diameter}}{\mathrm{Colony}\;\mathrm{diameter}}$$

Further, the quantitative estimation of soluble phosphate was carried out by using National Botanical Research Institute’s Phosphate Growth (NBRIP) medium (Nautiyal, 1999). The amount of soluble phosphate in culture free supernatant was determined by Fiske and Subbarow method (Fiske and Subbarow, 1925) and expressed as Soluble P (mg/L). Quantification of soluble phosphate was done through standard plot of KH_2_PO_4_.

### Optimization for Phosphate Solubilization by Selected ACC Deaminase Producing Isolates

The optimization study of phosphate solubilization was based on two attributes viz. incubation time and pH of the spent medium. The NBRIP medium was inoculated with overnight cultivated bacterial culture and incubated at 28°C in shaking incubator at 200 rpm. 5 ml bacterial culture was withdrawn at 24-h intervals to monitor the final pH of the medium and to evaluate efficiency of phosphate solubilization by ACC02 and ACC06 by Fiske and Subbarow method.

### Zinc Solubilization Assay of Selected Isolates

The zinc solubilizing potential of isolates was determined by spot inoculating the isolates in the Tris-minimal medium (per liter: Tris–HCl 6.06 g; NaCl 4.68 g; KCl 1.49 g; NH_4_Cl 1.07 g; Na_2_SO_4_ 0.43 g; MgCl_2_.2H_2_O 0.2 g; CaCl_2_.2H_2_O, 30 mg, pH 7.0; Fasim et al., 2002), supplemented with 1.5% agar and 0.1% (w/v) insoluble zinc in the form of zinc sulfate (ZnSO_4_) (Sharma et al., 2012). The plates were incubated for 14 days at 30°C and examined for the formation of halo zones around colonies for zinc solubilization.

### HCN, Ammonia, and Siderophore Production of Selected Isolates

For HCN production, the selected bacterial isolates were streaked on King’s B medium supplemented with 0.4% (w/v) glycine. A Whatman filter paper saturated with alkaline picric acid solution (2% Na_2_CO_3_ in 0.5% picric acid) was placed on the upper lids of Petri plates and monitored for 4 days for the development of red-brown from yellow color of filter paper, which served as the indicator for the HCN production (Miller and Higgins, 1970).

Estimation of ammonia was carried out by addition of Nessler’s reagent to bacterial culture in peptone water broth (peptone – 10 g/L; NaCl – 5 g/L) and development of slight yellow to brownish color (Kavamura et al., 2013).

The siderophore production was determined by the Chrome Azurol S (CAS) assay according to Schwyn and Neilands (1987). The bacterial colonies were spot inoculated on CAS agar plates and incubated at 28°C for 4 days. Development of orange-yellow halo around the growth was an indicator of siderophore producing bacterial isolates.

### *In vitro* Assay for Stress Tolerance in Response to Drought and Salinity

#### Salinity Stress Tolerance

For salt tolerance characterization, the growth of isolates was observed at 28°C for 72 h in the LB agar medium, supplemented with 2–8% NaCl concentration (Barra et al., 2016).

#### Drought Stress Tolerance

The drought tolerance of isolates was evaluated in polyethylene glycol (PEG 6000) supplemented LB agar medium. The growth of isolates was observed at 28°C for 24 h in LB agar media, with different osmotic potentials ranging from -0.05 to -0.73 MPa, and 25% polyethylene glycol (PEG 6000) was added to the medium for inducing drought stress (-0.73 MPa) (Ali et al., 2014).

### Effects of ACC Deaminase Producing Strains on French Bean Growth Under Salt Stress

The plant root elongation assay under normal watered and saline (25 mM NaCl) solution irrigated conditions was performed in accordance with Penrose et al. (2001) in an environmentally controlled growth chamber. Two putative ACC deaminase producing isolates, ACC02 and ACC06, were tested for their efficiency in augmenting growth and in alleviating the negative effects of salinity stress manifested on morphological parameters and chlorophyll content of French bean plants.

#### Preparation of Bacterial Inoculum Suspension

The selected bacterial inoculum was prepared in the DF salt minimal medium, supplemented with 3 mM ACC and incubated at 28°C in an orbital shaking incubator at 180 rpm for 3 days, in order to induce ACC deaminase activity of isolates. The cells were harvested at 12000 × g for 15 min and resultant cell pellet was suspended in 0.03M MgSO_4_ to achieve requisite concentration of cells (10^8^–10^9^ cfu/ml) at OD_600_. For preparation of microbial consortia, two bacterial strains must be compatible with each other in order to synergistically augment plant growth under non-stressed and salinity stressed conditions. The compatibility of two ACC deaminating rhizobacterial isolates, ACC02 and ACC06, was tested on nutrient agar medium by dual culture antagonism assay (Molina-Romero et al., 2017). The bacterial consortia were prepared by inoculating overnight grown bacterial suspensions of ACC02 and ACC06 strains in nutrient medium in the ratio of 1:1 (Saikia et al., 2018).

#### Sterilization and Bacterization of Seed

The seeds of French beans (*P. vulgaris* L.) variety (*A. Komal*) procured from Indian Agricultural Research Institute, New Delhi, India, were used for growth promoting experiments. They were surface sterilized in 70% (v/v) ethanol for 1 min followed by 10 min immersion in 1% (v/v) sodium hypochlorite solution (NaClO) and finally rinsed with sterile deionized water (6–7 times). For bacterial inoculation, the French bean seeds were inoculated for 1 h in the appropriate suspension of bacterial cultures and air dried aseptically in the laminar air flow. The control group comprised surface sterilized, unprimed French bean seeds, immersed in 0.03M MgSO_4_ solution.

#### Experimental Design and Salinity Treatment

The pot experimental trials were carried out in randomized block design (RBD) including four treatments in each normal and saline (25 mM NaCl) conditions for growth of French bean plants. The details of treatment under normal conditions in which plants were irrigated with distilled water without adding any external source of salt are shown in Table 1. The experimental design is similar in case of saline stress conditions but NaCl salt solution of EC 2.5 ds m^-1^ (25 mM NaCl), instead of distilled water, was used to irrigate French bean plants in order to artificially impose salinity stress (Table 1).

**Table 1:** Details of treatment for growth promotion assay of French bean plant by ACC02 and ACC06 in normal and saline conditions.

Growth conditions	Treatment no.	Seed treatment
Normal	T1; Treatment 1	Uninoculated seeds
	T2; Treatment 2	ACC02 bacterized seeds
	T3; Treatment 3	ACC06 bacterized seeds
	T4; Treatment 4	Consortium (ACC02 + ACC06) bacterized seeds
Saline (25 mM NaCl)	T1; Treatment 1	Uninoculated seeds
	T2; Treatment 2	ACC02 bacterized seeds
	T3; Treatment 3	ACC06 bacterized seeds
	T4; Treatment 4	Consortium (ACC02 + ACC06) bacterized seeds

#### Pot Experiment Assay

Three inoculated and uninoculated French bean seeds of respective treatment were then sown per plastic pots (30 cm in height and 30 cm in diameter), filled with autoclave-sterilized potting mixture of garden soil and coco peat in 1:1 ratio (3 kg soil pot^-1^). The experimental soil was characterized as sandy loam with pH 4.5, EC 0.0354 dS m^-1^, 66% sand, 9% slit, and 26% clay (Table 2). The pots were placed in a growth chamber and maintained under optimum light and temperature condition, i.e., 80% relative humidity, 16:8 light: dark photoperiod and at 25°C for 30 days. After 10 days of seedling emergence, French bean seedlings were irrigated daily, twice a day, with either sterile distilled water or solution of EC 2.5 ds m^-1^ (25 mM NaCl; to artificially induce salinity stress) as per the treatment condition. The unbacterized plants subjected to salinity stress were presented as a negative control group while non-saline, unbacterized plants served as positive control group.

**Table 2:** Physio-chemical characteristics of experimental soil.

Texture	pH	EC (ds m^-1^)	Organic C (g kg^-1^)	Total N (g kg^-1^)	Total P (g kg^-1^)	Total K (g kg^-1^)
Sandy loam	4.5	0.0354	0.58	0.19	0.02	0.23

### Morphological and Physiological Analysis of Plants

French bean plants, after being grown for 20 days under saline and non-saline condition, were harvested (three plants per treatment) and analyzed for growth related parameters including root/shoot length, root/shoot fresh and dry weight. The roots and shoots were oven dried separately at 60°C for 3 days and weighed to determine dry biomass of French bean plants. The leaf chlorophyll content was measured by UV/Vis spectrophotometer at 645 and 663 nm in accordance with (Lorenzen, 1967; Hiscox and Israelstam, 1979). In response to salinity stress, electrical conductivity of soil suspension (1:4) of respective treatment was recorded at the stage of harvesting.

### Ethylene Measurement

The ethylene emission from French bean seedlings of each treatment was determined by following the protocol of Sarkar et al. (2018), in order to evaluate the potential of ACC deaminase producing strains ACC02 and ACC06 in lowering stress generated ethylene production. The French bean seedlings (three seedlings per treatment) were placed inside the 60-mL glass tubes, sealed with rubber septum and incubated at 28°C for 4 h. One milliliter of headspace gas was withdrawn and injected into the Gas Chromatograph (Bruker 450-GC, Bruker Corporation, United States) equipped with flame ionization detector (FID). The amount of ethylene evolved was expressed as micromole of ethylene per gram of fresh weight by comparing with the standard curve of pure ethylene.

### Molecular Identification of Rhizobacterial Isolates

#### DNA Isolation and 16S rRNA Gene Sequencing

The genomic DNA of two potent ACC deaminase producing PGPR was extracted in accordance with Pandey et al. (2013). The isolated genomic DNA from strains was analyzed on 0.8% agarose gel and amplification of 16S rRNA (∼1500 bp) was carried out in a polymerase chain reaction (PCR) using universal primers forward (5′AGAGTTTGATCCTGGCTCAG3′) and reverse (5′AAGGAGGTGATCCAGCCGCA3′).

The 25 μL reaction mixture for PCR amplification was composed of PCR Buffer 2.5 μL; MgCl2 2 μL; 2 mM dNTPs 1 μL; Primers 0.5 μL each; Taq DNA polymerase 0.5 μL; Template DNA 2 μL; Sterile deionized water 16 μL. The PCR amplified product was purified with Qiaquick PCR purification kit (Qiagen, Valencia, CA).

#### Phylogenetic Characterization

The 16S rRNA gene was sequenced by Eurofins Genomics India Pvt. Ltd. (Bengaluru, India) by Sanger’s di-deoxy nucleotide sequencing method. A similarity search for the sequence so generated was performed using National Centre of Biotechnology Information (NCBI) BLAST program^1^. The phylogenetic tree was constructed by Neighbour-joining (NJ) method using software MEGA X with the bootstrap of 1000 replicates and evolutionary distances were computed.

#### Nucleotide Accession Number

The 16S rRNA gene sequences so obtained in the present study were submitted to NCBI GenBank database and assigned the accession numbers MH645748 and MH645749 for isolates ACC2 and ACC6, respectively.

### Statistical Analysis

All the data regarding quantitative estimation of PGP traits were subjected to one-way ANOVA followed by Tukey’s test. All the statistical analyses were carried out with help of SPSS software. The experiments were performed in three replicates and the mean, as well as standard deviation, were calculated using Microsoft Excel 2016.

## Results

### Bacterial Isolation and Preliminary Assessment of ACC Deaminase Activity

A total of twenty rhizobacteria were isolated from rhizospheric soil of *A. sativum* on enrichment media, of which, six strains were able to grow on DF minimal salt medium supplemented with 3 mM ACC as a nitrogen source, implying ACC deaminase activity.

The ACC deaminase activity of these six isolates, labeled as ACC02, ACC04, ACC06, ACC07, ACC011, and ACC012, was further quantified in terms of α-ketobutyrate production.

### Quantitative Estimation of ACC Deaminase Activity

**FIGURE 1:**
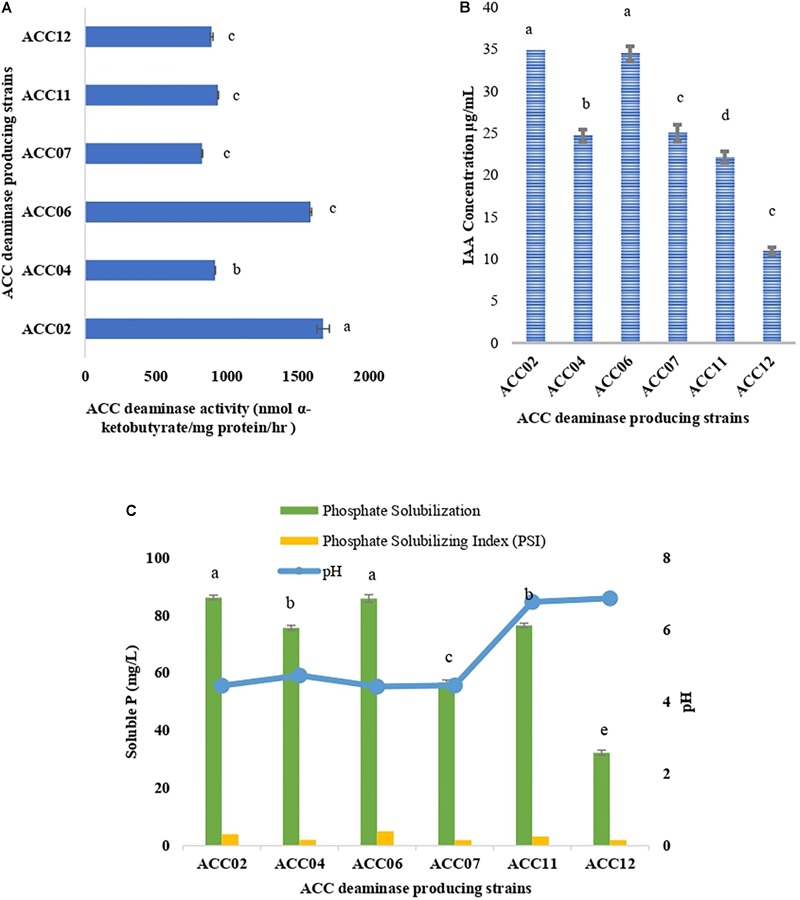
Quantitative estimation of **(A)** ACC deaminase activity, **(B)** IAA production, and **(C)** phosphate solubilization of six isolates from *Allium sativum* rhizospheric soil in respective DF minimal salt, LB-trp, and NBRIP medium. Columns represent mean values while bars represent standard deviation (*n* = 3). Different letters show statistically significant different values (*P* < 0.05) from each other as evaluated from Turkey’s test.

**FIGURE 2:**
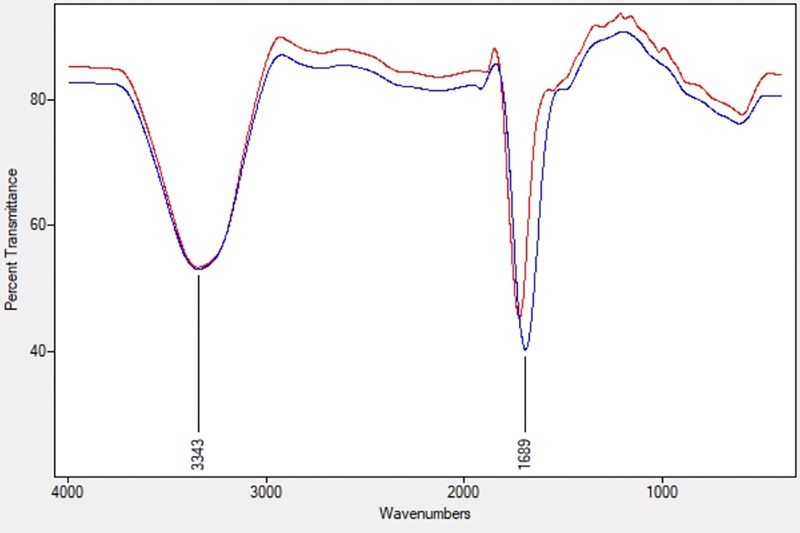
FTIR spectra of α-ketobutyrate in the strain ACC02 (red; upper line) and ACC06 (blue; lower line).

The ACC deaminase activity of isolates was quantified by α-ketobutyrate production by catalyzing the deamination reaction of sole nitrogen source, ACC in DF minimal salt broth media at 540 nm. All the test isolates showed variation in ACC deaminase activity in the range of 900–1800 nmol α-ketobutyrate per mg of cellular protein per hour. The highest ACC deaminase activity was exhibited by bacterial strain ACC02 (1677 nmol α-ketobutyrate mg protein^-1^ h^-1^) followed by ACC06 (1589 nmol α-ketobutyrate mg protein^-1^ h^-1^), ACC11 (936 nmol α-ketobutyrate mg protein^-1^ h^-1^), ACC04 (916 nmol α-ketobutyrate mg protein^-1^ h^-1^), ACC12 (891 nmol α-ketobutyrate mg protein^-1^ h^-1^) and ACC07 (824 nmol α-ketobutyrate mg protein^-1^ h^-1^) (Figure 1A). The highest enzymatic activity of ACC deaminase produced by ACC02 and ACC06, i.e., conversion of nitrogen source ACC into α-ketobutyrate, was further verified by FTIR spectra analysis results (Figure 2), which showed peaks at 1689 and 3343 cm^-1^, confirming the presence of a ketonic group and amino functional group, respectively recognized as α-ketobutyrate as per Sarkar et al. (2018).

### Quantitative Determination of Indole Acetic Acid Production

The production of IAA by six putative ACC deaminase producing isolates was quantified at 530 nm by supplementing the growth media with L-tryptophan. The IAA production of all isolates ranged between 10.96 and 37.78 μg/mL with ACC02 (37.38 μg/mL) isolate being displayed as the highest IAA producer followed by isolate ACC06 (34.48 μg/mL) (Figure 1B).

**FIGURE 3:**
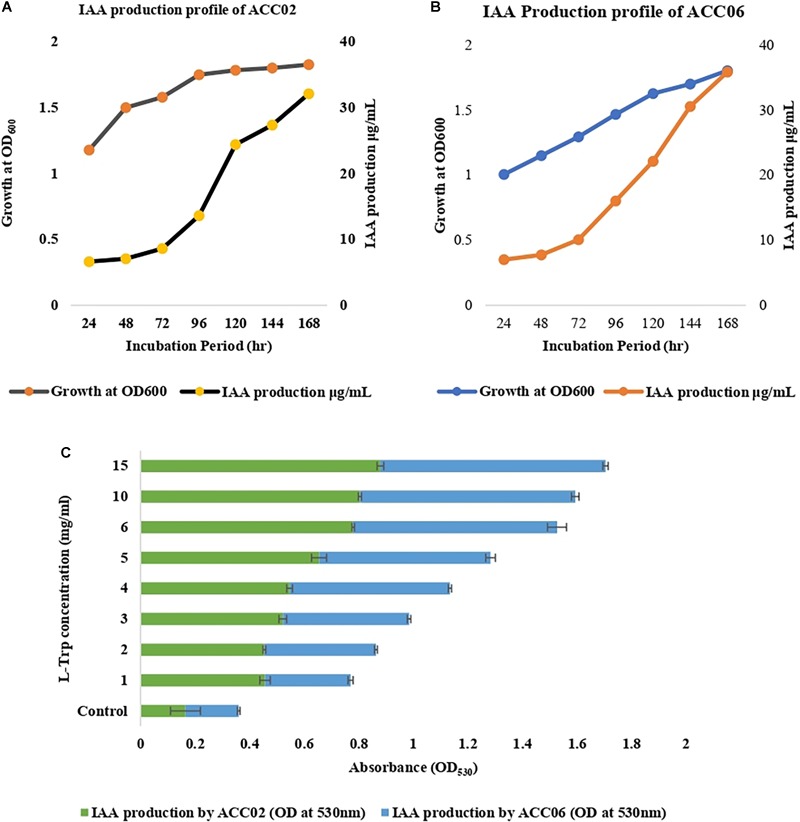
IAA production profile of ACC02 **(A)** and ACC06 **(B)** isolates as a function of incubation period and of L-tryptophan concentration **(C)**.

The isolates showing positive results with respect to others, ACC02 and ACC06, were further selected for optimization studies to study the effects of incubation period (Figure 3A,B) and substrate, L-tryptophan concentration (ranging from 1 to 15 mg/mL) on IAA production (Figure 3C). In the time course of IAA production, the maximum production of IAA by both the putative ACC deaminase producers, ACC02 and ACC06, was found on the 7th day of the incubation period. According to our results, the maximum IAA production was found when the media was supplemented with L-tryptophan concentration (15 mg/mL) in comparison to unamended media (without L-trp).

### Phosphate Solubilization

**FIGURE 4:**
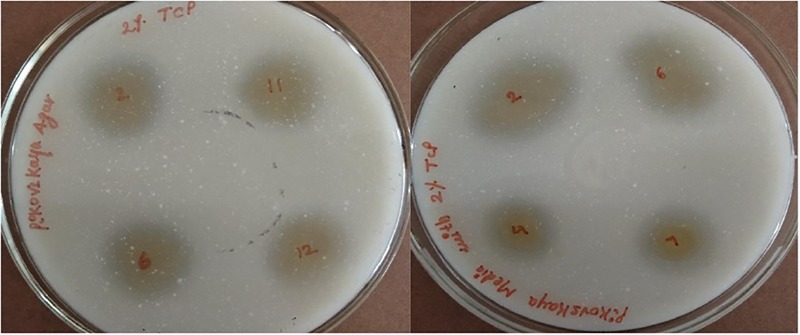
Phosphate solubilization by different isolates from *Allium sativum* rhizospheric soil on Pikovaskya’s agar supplemented with 2% TCP.

The potential of six ACC deaminase producing isolates to convert inorganic forms of phosphorous TCP into the solubilized form was assessed when the yellow color zone of phosphate solubilization was displayed around the colonies on Pikovaskya’s agar supplemented with 2% TCP (Figure 4). The maximum solubilization index was exhibited by isolates ACC02 and ACC06 (>3).

These calcium phosphate solublizers were further analyzed quantitatively in NBRIP medium as shown in Figure 7, where the amount of soluble phosphate was within the range of 30–88 mg/L P. The highest phosphate solubilization of 86.25 mg/L was shown by ACC02 followed by ACC06 and ACC11. The result observed indicates that isolates showing maximum zone of solubilization on solid medium, are also showing maximum phosphate solubilization in liquid medium. The pH of NBRIP medium was found to be acidic after 7 days of incubation as compared to uninoculated control (pH 6.5), except in the case of two isolates, ACC11 and ACC12 (6.7 and 6.8, respectively) (Figure 1C).

**FIGURE 5:**
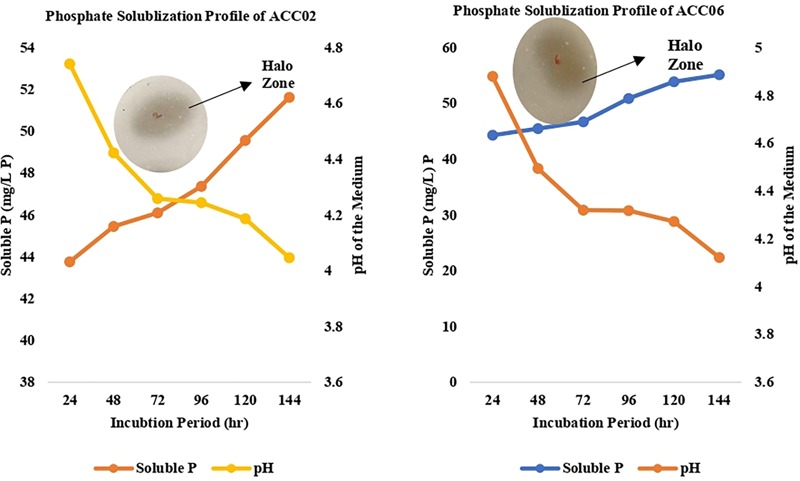
Calcium phosphate solubilization profile of ACC02 and ACC06 isolates as a function of incubation period and pH of spent medium.

The two strains ACC02 and ACC06 with highest phosphate solubilizing ability were further selected for determining the profile of phosphate solubilization with respect to incubation period and pH of the NBRIP medium. The highest solubilization was recorded on the 6th day of incubation for both the isolates, ACC02 and ACC06. Further, the optimization studies also represented that both the isolates lowered the pH of broth medium as the incubation period increases with respect to uninoculated control medium (Figure 5). The lowering of pH of medium, attributed by the production of organic acids, was in correlation with P solubilizing activity, with lowest pH recorded during maximum calcium phosphate solubilization.

### Production of Ammonia, HCN, Siderophore, and Solubilization of Zinc

**FIGURE 6:**
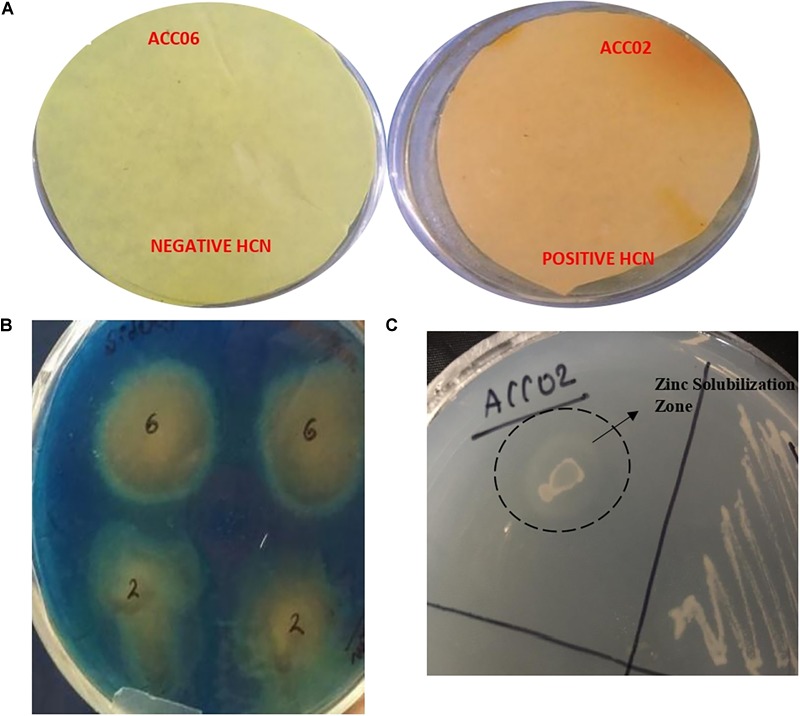
Estimation of HCN production **(A)**, Siderophore production on solid blue CAS agar media **(B)**, and zinc solubilization on Tris minimal salts agar medium supplemented with ZnSO_4_ **(C)** by ACC deaminase producing isolates ACC02 and ACC06.

The HCN (hydrogen cyanide) production was observed for isolate ACC02 (*A. aneurinilyticus* AIOA1), while the yellow picrate filter paper did not turn red brown for other isolate ACC06 (*Paenibacillus* sp. strain SG_AIOA2) as shown in Figure 6A. Another indirect PGP mechanism, Ammonia (NH_3_) production, was checked for both the potent ACC deaminase producers. Both the isolates ACC02 (*A. aneurinilyticus* AIOA1) and ACC06 (*Paenibacillus* sp. strain SG_AIOA2) were positive for ammonia production. In addition to this, both isolates exhibited a color change of greenish blue CAS agar media to yellow indicating significant production of siderophore (Figure 6B). The Zinc solubilization ability of the ACC deaminase producing bacterial isolates were evaluated by determining zone of solubilization on ZnSO_4_ supplemented Tris-minimal medium. The isolate ACC06 did not solubilize zinc while another isolate ACC02 produced halo zone (Figure 6C).

### *In vitro* Stress Tolerance in Response to Salinity and Drought

In addition to this, both the isolates were screened *in vitro* for their potential to tolerate high salt concentration (2–8%) and osmotic stress conditions. In the case of salinity, the isolate ACC02 could be able to tolerate salinity levels up to 6% NaCl while the isolate ACC06 exhibited growth on LB media supplemented with 4% NaCl only. No isolates could not tolerate high concentration of NaCl (8%) added in growth media.

The ACC02 isolate was able to tolerate the osmotic stress of 0.05 MPa only and not higher drought conditions, while ACC06 could survive in drought conditions of 0.05 MPa, 0.30 MPa as well as 0.73 MPa osmotic stress.

### Molecular Identification and Phylogenetic Analysis

**FIGURE 7:**
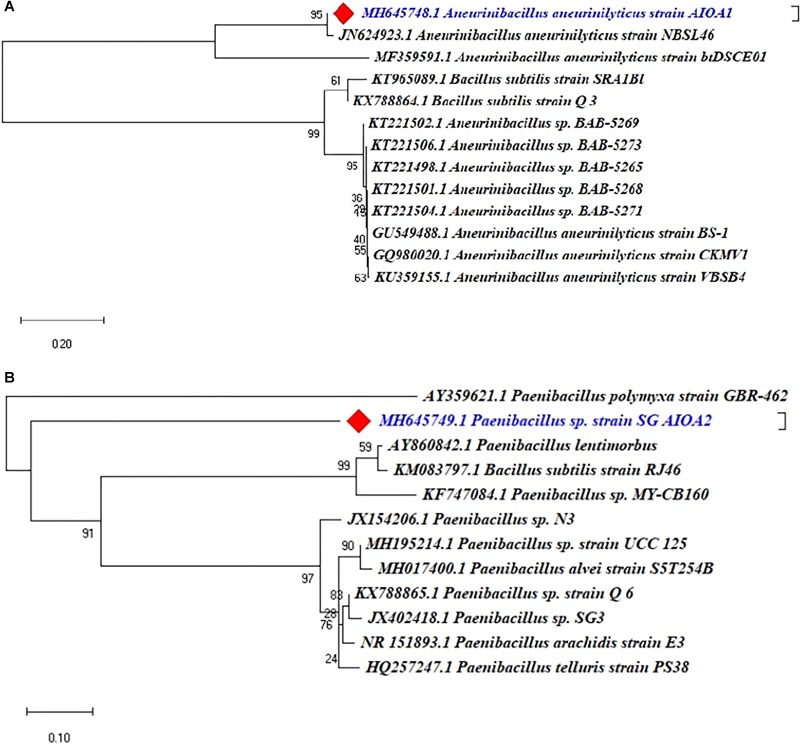
Phylogenetic dendrograms, based on partial 16S rRNA nucleotide sequences, showing the relationship between the selected ACC deaminase producing strains ACC02 and ACC06 with closely related taxa of **(A)** *Aneurinibacillus* and **(B)** *Paenibacillus*, respectively. The 16S rRNA gene sequences of closely related species were retrieved from NCBI GenBank databases. The Neighbour joining phylogenetic tree was inferred using MEGA-X software; evolutionary distance was computed using Maximum Composite Likelihood method at bootstrap value of 1000.

The 16S rRNA gene sequencing and phylogenetic analysis revealed that two ACC deaminase producing PGPR isolates indicated 95–100% similarity with the known sequences in GenBank and belonged to different genera of *Aneurinibacillus* and *Paenibacillus*. The Phylogenetic analysis of two ACC deaminase producing strains using MEGA X software revealed their relatedness with other strains of respective species (Figure 7).

### Effects of ACC Deaminase Producing Strains on French Bean Growth Under Salt Stress

**FIGURE 8:**
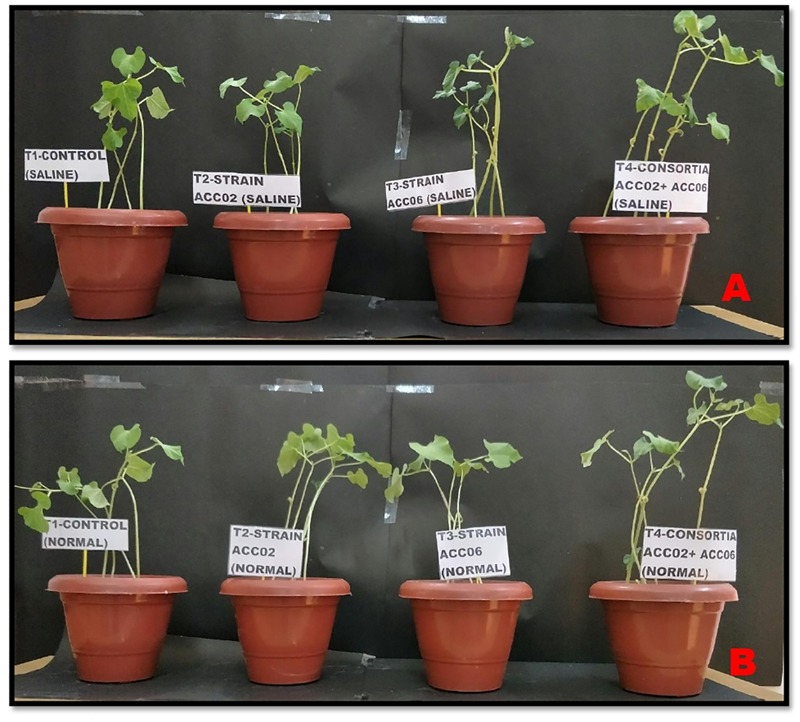
Effect of ACC deaminase producers, ACC02 and ACC06, as individual strains and consortium on plant growth promotion of French bean under salinity stress **(A)** and normal condition **(B)**, as compared to positive (uninoculated plants growing in normal conditions) and negative control (uninoculated plants growing saline stress conditions).

The influence of the two highest ACC deaminase, IAA producing and Phosphate solubilizing PGPR strains, ACC02 and ACC06, on growth promotion of French bean under no stress and salinity stress conditions, was evaluated through pot trials (Figure 8). The dual culture antagonism assay between these two selected strains did not show any antagonistic effects against each other (Supplementary Figure 1).

The negative effects of salinity stress resulted in a reduction in growth parameters such as root length, shoot length, fresh and dry biomass of root and shoot of French bean plants in relation to corresponding non-stressed, uninoculated French bean plants. The EC of the experimental soil of all salt treated pots was recorded in the range of 4–4.8 ds m^-1^.

However, the plant growth in terms of growth parameters, including length, fresh and dry weight of root/shoot biomass, was significantly (*P*< 0.05) increased in bacterial inoculated plants under salinity stressed and non-saline conditions.

**FIGURE 9:**
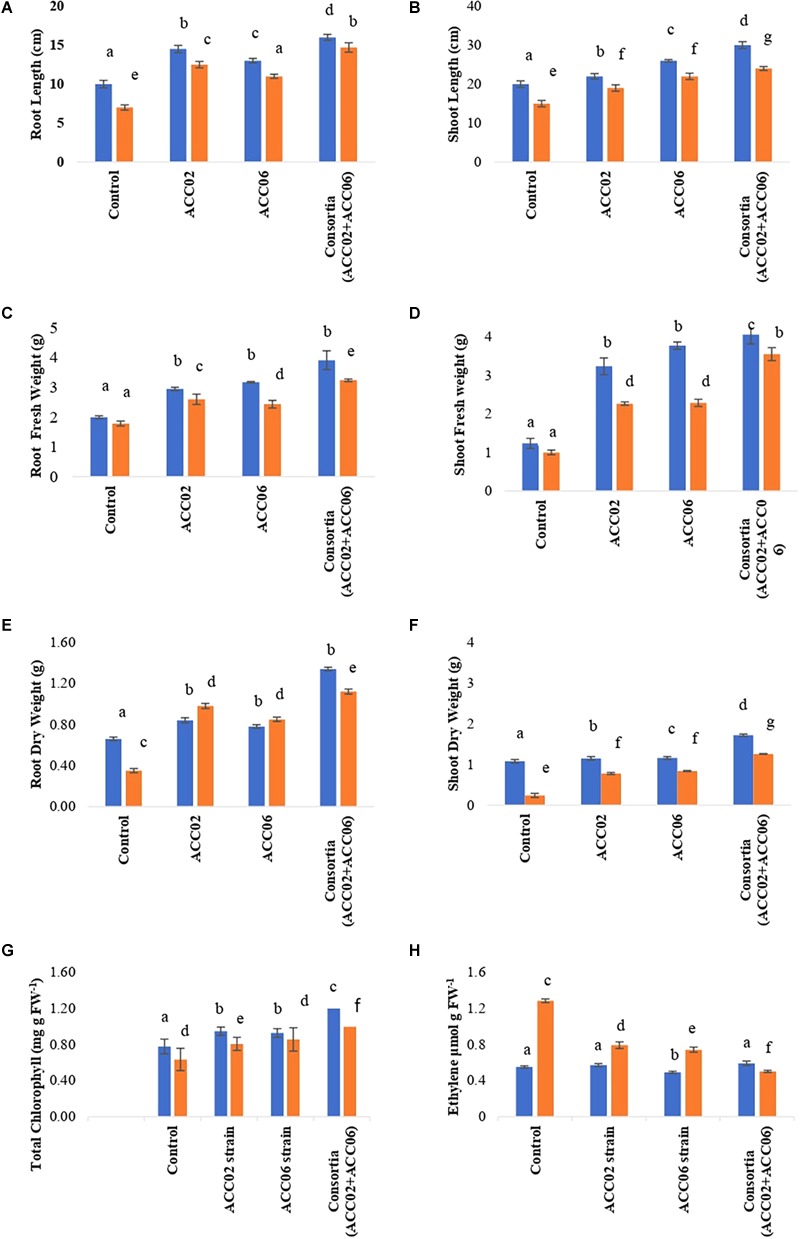
Effect of potent ACC deaminase isolates on physio-morphology parameters: **(A)** Root Length; **(B)** Shoot Length; **(C)** Root Fresh weight; **(D)** Shoot Fresh weight; **(E)** Root Dry weight; **(F)** Shoot Dry Weight; **(G)** Total Chlorophyll content and **(H)** Ethylene content of French beans plants under stress (saline; orange bar) and non-stressed (normal; blue bar) conditions. Control, plants from unbacterized seeds; ACC02, seeds inoculated with *A. aneurinilyticus* strain AIOA1; ACC06, seeds inoculated with *Paenibacillus* sp. strain SG_AIOA2. Columns represent Mean values while bars represent Standard deviation (*n* = 3). Different letters show statistically significant different values (*P* < 0.05) between treatments as evaluated from Duncan’s test.

The consortium application of ACC02 and ACC06 enhanced the growth of root and shoot of French bean plant exposed to salt stressed conditions. The shoot length was significantly (*P*< 0.05) improved by 60% in T4 treatment in comparison with corresponding uninoculated control plants. However, no significant difference was found in the case of shoot length between individual strain application, i.e., T2 and T3 treated French plants (Figure 9B). Similarly, the combined treatment of ACC deaminase producing strains, ACC02 and ACC06, has a tremendous influence on root length of French bean plants on exposure to salt stressed conditions. The root length was increased by ∼79, 58, and 110% in T2, T3, and T4 treatments as compared to control stressed group of plants (Figure 9A).

The consortium treatment improved the fresh and dry biomass of root/shoot of French bean plants under saline stress conditions. The shoot fresh weight and dry weight of French bean plants increased by ∼255 and ∼425% under saline stress conditions in relation to corresponding control. No considerable difference was found in the case of shoot biomass of French plants treated with individual strains ACC02 and ACC06. Similar improvements were achieved in terms of root fresh weight and dry weight of French bean plants under salt stress. Root fresh weight significantly (*P*< 0.05) increased by ∼46, 36, and 81%, while Root dry weight improved by 180, 142, and 220% at T2, T3, and T4 treatments with respect to T1 treated control plants (Figure 9C–F).

The consortium treatment was found to be effective in improving the leaf chlorophyll content of stressed French bean plants by ∼57%, followed by ∼35% at Treatment T3 and ∼28% at treatment T2, in comparison with the uninoculated stressed plants (Figure 9G). The higher levels of ethylene production ∼133% was observed in salt stressed uninoculated French bean plants, in comparison to plants grown in normal (0 mM NaCl) conditions. However, inoculation of French bean plants with ACC deaminase producers, *A. aneurinilyticus* and *Paenibacillus* sp., reduced the ethylene emission by 38 and 42%, respectively, compared with uninoculated French bean plants. The consortium application of *A. aneurinilyticus* and *Paenibacillus* sp. has more potential in decreasing salinity stress induced ethylene by ∼61% followed by treatment 2 and treatment 3 in inoculated plants (Figure 9H).

These results suggested that consortia of isolates *A. aneurinilyticus* (ACC02) and *Paenibacillus* sp. (ACC06) have the potential to mitigate the negative effects of salt stress on the growth of French bean plants.

## Discussion

The possession of enzymatic activity of ACC deaminase by PGPR is one of the key growth stimulating traits that facilitate growth and stress tolerance in plants under normal as well as stressed conditions (Glick, 2012; Chandra et al., 2018). This microbial enzyme, ACC deaminase, is responsible for dissociation of stress induced ACC (secreted as root exudates) into Ammonia and α-ketobutyrate, which otherwise forwarded to produce ethylene that has a drastic impact on physiology, growth and development of plants (Barnawal et al., 2014; Heydarian et al., 2016; Ali and Kim, 2018; Saleem et al., 2018; Zhang et al., 2018). Therefore, the role of microbial ACC deaminase producers is immensely significant in today’s agricultural system, which is more prone to climate change.

In the present work, two bacterial isolates ACC02 and ACC06 with a high amount of ACC deaminase activity were selected from the rhizosphere of *A. sativum* crop plant for further assessment for growth promoting abilities such as IAA production, insoluble phosphate and zinc solubilization, siderophore secretion, HCN and ammonia production.

The PGPR altered the indigenous pool of growth regulating hormones, such as IAA, which resulted in root elongation as well as formation of lateral roots and root hairs. This enhancement of root system of plants improves water and nutrient uptake efficiency of plants. The endogenous IAA along with bacterial synthesized IAA increased the secretion of plant root exudates that directly serve as an energy source for root associated growth promoting bacteria, improving their growth and colonization efficiency (Etesami et al., 2015). Several strains of genus *Bacillus, Azotobacter, Pseudomonas* were reported to produce IAA (Cassán et al., 2014; Verma et al., 2018). The evaluation of bacterial isolates, ACC02 and ACC06 for production of IAA, revealed that both are significant producers of IAA suggesting that they could be used as PGPR.

The optimization process is very important as it helps in optimizing the attributes, such as the minimum number of days as well as optimum precursor, L-tryptophan concentration responsible for maximum IAA production by PGPR. The optimization of IAA production by these two isolates indicated highest production after 7th day of inoculation. A similar result was reported by Chaiharn and Lumyong (2011), indicating maximum IAA production on the 9th day of inoculation. The L-tryptophan is observed as the direct precursor for IAA synthesis by root colonizing bacteria through the indole-3-acetamide pathway (Patten and Glick, 1996), therefore, the amount of precursor concentration has a direct influence on IAA production. As per our study, growth media amended with different concentrations of L-tryptophan was used to investigate the effect of substrate concentration on IAA production. On the basis of spectrophotometric data (OD_530_), it was evaluated that IAA production was increased with an increasing concentration of externally supplied L-tryptophan with maximum production in medium supplemented with 15 mg/mL of tryptophan. Similar results were observed by Bharucha et al. (2013) who demonstrated the influence of L-tryptophan concentration on indole production and found a positive correlation between IAA production and L-tryptophan concentration.

Phosphate solubilization is another important trait of PGPR and the isolates of the current study revealed a significant release of PO_4_^3-^ from insoluble complex TCP. The findings of the current study are in agreement with numerous published literatures reporting solubilization of phosphate by *Bacillus, Pseudomonas*, and *Azotobacter* (Ahmad et al., 2008; Bhattacharyya and Jha, 2012; Panwar et al., 2014; Maitra et al., 2015). Besides providing phosphorous to the plants, the PSBs also enhance the plant’s growth by stimulating nitrogen fixation, enhancing availability of trace metals and by formation of plant growth hormones (Kumar et al., 2013). It has been reported that quantitative measurement of P-solubilization in NBRIP broth medium provides a more accurate result than regular plate assay (Baig et al., 2010). Therefore, evaluation of isolates to solubilize phosphorous in NBRIP broth was also completed, which revealed direct co-relation between P-solubilization on plates and broth medium. The P solubilization by these two isolates was accompanied by a decrease in pH of the medium, indicating the mechanism behind P-solubilization is secretion of organic acids, such as gluconic acid and citric acid, by the isolates (Serrano et al., 2013; Khan et al., 2014; Chen et al., 2016; Paul and Sinha, 2017; Wei et al., 2018). Furthermore, the optimization analysis revealed that phosphate solubilizing efficiency of isolates, ACC02 and ACC06, progressively increased with the number of incubation days. The maximum phosphate solubilization in NBRIP medium was observed at the 6th day of incubation for both isolates, followed by a decline in the pH of the medium. The results of optimization of phosphate solubilizing potential of our isolates are in agreement with the similar work of Manzoor et al. (2017).

The production of siderophore, ammonia and HCN are some of the indirect PGP traits found among the isolates of the present study. Siderophores, low molecular weight iron chelators, produced by various soil microorganisms, bind Fe^3+^ and make it available for their own growth and also for plants. There are several reports describing the production of siderophores by the rhizospheric micro flora enhancing the iron uptake of plants (Kotasthane et al., 2017; Priyanka et al., 2017).

The production of ammonia by the microbes helps the plants both directly and indirectly. The ammonia excreted by diazotrophic bacteria is one of the most important characters of the PGPR’s which benefits the crop (Pérez-Montaño et al., 2014; Richard et al., 2018). This accumulation of ammonia in soil may increase in pH creating alkaline condition of soil at pH 9–9.5. It suppresses the growth of certain fungi and nitrobacteria as it has potent inhibition effect. It also disturbs the equilibrium of the microbial community and inhibits germination of spores of many fungi (Swamy et al., 2016).

Zinc is a crucial micronutrient for growth and development of plants governing several their physiological processes. Use of zinc solubilizers in place of chemical fertilizers is an eco-friendly and sustainable method to meet the requirements of zinc by the plants (Kamran et al., 2017). The zinc solubilizing ability of ACC deaminase containing PGPR was evaluated on the Tris-minimal plates supplemented with insoluble zinc complex, ZnSO_4_, and only ACC02 was found to be categorized as zinc solubilizing bacteria.

The tolerance of bacteria toward high salinity and drought was also screened under *in vitro* conditions. Both isolates indicated moderate tolerance toward salt and drought stress. Therefore, isolates ACC02 and ACC06 have could be used as stress tolerating PGPR in soils facing challenges of salinity and drought.

The BLAST similarity searches and phylogenetic analysis revealed that isolates of our study, ACC02 and ACC06, belonged to *A. aneurinilyticus* strain AIOA1 and *Paenibacillus* sp. strain SG_AIOA2, respectively. There are few reports available in the literature documenting the ACC deaminase producing ability and PGPR potential of *Aneurinibacillus* and *Paenibacillus* (Goswami et al., 2015; Chauhan et al., 2017).

The present study reports that inoculation of seeds with ACC deaminase producing bacteria reduces the stress of salinity in plants. The results of *in vivo* experiments also reveal that there is a significant improvement in plant growth of the test plant under both no salt and salt stressed conditions when both strains were applied as consortium compared to application of individual strains. The finding dual culture antagonism assay as well as pot study reveals that both strains were compatible to each other and exhibited their cumulative growth promotion effect when applied together. The interaction between different microbial strains result into enhanced PGP potential of PGPR. It has been reported that the syntrophic relationship among microbes is a common phenomenon in microbial ecosystem (Morris et al., 2013). Thus, use of consortia of microbes having synergistic relation is more beneficial and exerts improved results (Shaharoona et al., 2006; Verma et al., 2013). The presence of a higher number of bacterial cells in a consortium could be the reason behind greater PGP effects under stressed conditions (Barra et al., 2016; Grobelak et al., 2018; Saikia et al., 2018).

The ethylene is a significant plant hormone required for every aspect of plant development and growth cycle but it stimulates, growth repressive effects, such as retardation of root development, inhibition of seed germination, promotion of aging, senescence and abscission process in plants under stressed conditions (Gupta and Pandey, 2019). Several reports suggested that application of ACC deaminase producers mitigate the adverse effect of salt stress manifested on plants by minimizing the ethylene emission to its optimum level and thus confers growth promotion and stress tolerance in stressed plants (Orhan, 2016; Maxton et al., 2018; Safari et al., 2018). The growth of French bean plants when subjected to salinity stress might be attributed to the ACC deaminase activity (>20 nmol α-ketobutyrate mg protein^-1^ h^-1^) of two selected strains, which declines the production of stress generated ethylene by deaminating its precursors, ACC into ammonia and α-ketobutyrate (Penrose and Glick, 2003). Based on our results and previous findings, ACC utilizing bacterial strains promote plant growth, increase root/shoot length and improve plant biomass under salinity stress conditions by lowering ethylene accumulation in plants. However, the presence of multifarious PGP traits could be the added advantage of these isolates, which could be harnessed to develop these strains as bio-fertilizers in a wide range of plants under normal conditions. Our study suggests that the application of a consortia of microbes enhances plant growth in both normal and saline conditions. It is very much possible that IAA and ACC deaminase promote root growth in a co-ordinated manner (Glick et al., 2007). Similarly, siderophore production and phosphate solubilization has also been reported by various researchers as an important attribute significantly responsible for plant growth and development (Rajkumar et al., 2010; Taurian et al., 2010; Sadeghi et al., 2012; Zhang et al., 2017; Kumar et al., 2018; Patel et al., 2018). Therefore, use of microbial consortia having the potential to induce salt tolerance and also enhance plant growth and development under normal condition will be a very useful strategy in sustainable agriculture. However, further research is needed to evaluate the efficiency of these strains as their consortium under field condition to provide tolerance in the case of abiotic stress like salinity and exertion of their PGP potential under normal field condition.

## Conclusion

The present study describes the isolation of ACC deaminase producing bacteria from rhizospheric soil of Garlic. Two potential strains, ACC02 and ACC06, were found to possess other growth promoting potential like IAA production, phosphate solubilization, siderophore and ammonia production. The result of *in vivo* study of these isolates revealed that they promote plant growth both under normal and saline conditions. The production of ACC deaminase and other PGP traits by these isolates project the potential that they could be used as a bio-fertilizer under both normal and saline soils. However, further research is needed to evaluate the efficiency of these strains under actual field conditions to reduce the stress of salinity and as an effective plant growth promoter.

## Author Contributions

SG has conducted all the experiments under the guidance of SP. SP conceived and designed the research. Both the authors analyzed the data and wrote manuscript.

## Conflict of Interest Statement

The authors declare that the research was conducted in the absence of any commercial or financial relationships that could be construed as a potential conflict of interest.

## Footnotes

1 https://blast.ncbi.nlm.nih.gov/Blast.cgi
