# Supplementary figures and images for: ACC Deaminase Producing Bacteria With Multifarious Plant Growth Promoting Traits Alleviates Salinity Stress in French Bean (Phaseolus vulgaris) Plants
Source: Front Microbiol. 2019 Jul 9;10:1506. doi: 10.3389/fmicb.2019.01506 (PMC6629829; doi:10.3389/fmicb.2019.01506)

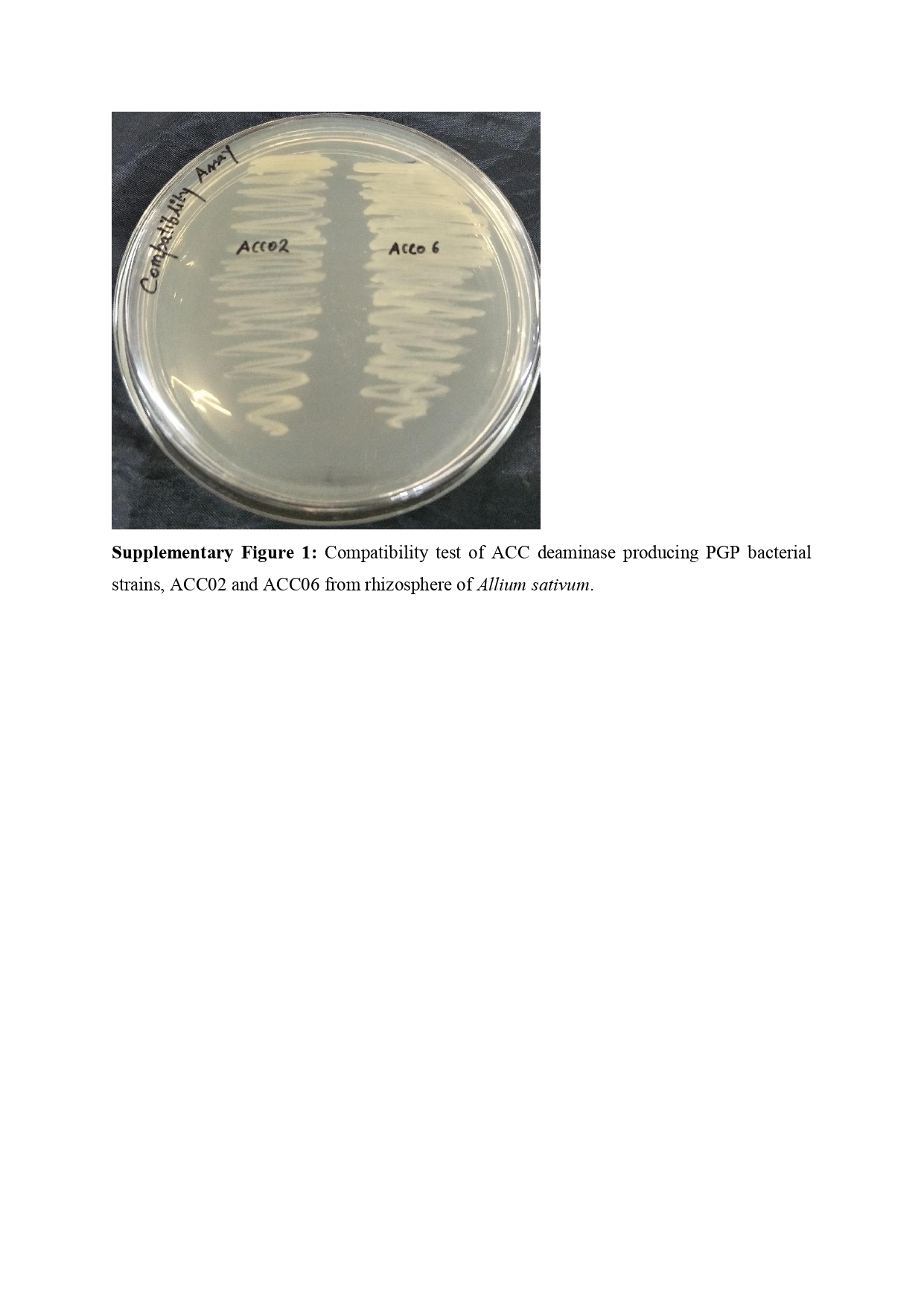

Supplement: Supplementary file 1 [file Image_1.JPEG]
